# Efficacy of sequential N-butylphthalide therapy on psychiatric and behavioral functions in acute ischemic stroke

**DOI:** 10.1097/MD.0000000000027860

**Published:** 2021-11-19

**Authors:** Le Yang, Hui Li, Yanzhi Wu, Hongdan Zhang, Jieqiong Du, Yankun Chen

**Affiliations:** a Department of Neurology, the First Affiliated Hospital of Guangxi Medical University, Nanning City, Guangxi Province, China; b Department of Urologic, Heze Municipal Hospital, Heze City, Shandong Province, China; c Department of Gastroenterology, Heze Municipal Hospital, Heze City, Shandong Province, China; d Department of Intensive Care Unit, Heze Municipal Hospital, Heze City, Shandong Province, China; e Department of Neurology, Heze Municipal Hospital, Heze City, Shandong Province, China.

**Keywords:** butylphthalide, NBP, sequential therapy, stroke

## Abstract

**Background:**

Stroke can cause physical and mental problems. This study examined how the sequential therapy of N-butylphthalide (NBP) could effectively improve physical movement, life activities, and psychological disorders in stroke patients.

**Methods:**

This double-blind, randomized controlled trial included middle-aged or elderly patients with acute ischemic stroke that had commenced within 48 hours before enrolment in the study. The experimental group was administered 100 mL NBP injections twice a day in the first 14 days, and a sequential 200 mg NBP soft capsule 3 times a day for the next 76 days. The control group was administered 100 mL NBP placebo injections twice a day in the first 14 days and 200 mg sequential NBP placebo soft capsule 3 times a day for the next 76 days. Primary outcomes were the National Institutes of Health Stroke Scale, the Barthel Index of activities of daily living, and Modified Rankin Scale which were evaluated at day 0, day 14, and month 1 or at day 14, month 3, and month 6. Secondary outcomes included the Hamilton Anxiety Scale and the Hamilton Depression Scale, all were evaluated on day 0, month 3, and month 6. Moreover, the adverse reaction of NBP or other serious adverse events were evaluated at each time.

**Results:**

Our therapy significantly increased the Barthel Index of activities of daily living scores, decreased the National Institutes of Health Stroke Scale and Modified Rankin Scale scores, and the incidence of the Hamilton Anxiety Scale and the Hamilton Depression Scale of ischemic stroke patients (*P* < .05).

**Conclusion:**

Our results indicated that 90 days’ sequential therapy with NBP as an additional therapy in the treatment of ischemic stroke can better improve patients’ psychological and behavioral functions without significant side effects.

## 1 Introduction

Stroke is the most common cause of disability globally, the second leading cause of death,^[1]^ and the third leading cause of disability worldwide.^[2]^ In China, stroke has been reported as the leading cause of death in urban and rural areas.^[3,4]^ Stroke can lead to a series of disorders that involve motor function, activities of daily living, cognition, and psychology. The incidence of psychological and cognitive disorders is very high, and the long-term cognitive impact of stroke on patients is more than that of physical disorders. Studies have confirmed that the prevalence of cognitive impairment in stroke patients is 50% to 70%,^[5]^ and the prevalence of poststroke depression (PSD) ranges from 30% to 65%.^[6]^ Conversely, poststroke anxiety (PSA) has only recently been studied.^[7,8]^ The comorbidities of PSA and PSD are very high, with up to 85% of patients developing anxiety and depression within 3 years after stroke.^[9,10]^ This has greatly affected patients’ quality of life and confidence in returning to societies, and has brought a heavy economic and psychological burden on families and societies, thereby making it a considerable public health problem.^[11]^

In China, stroke units experienced an increasing use of proprietary Chinese medicine to adapt to the growing needs of patients. Stroke rehabilitation has gradually developed into a clinical model of integrated traditional Chinese and Western medicine. After artemisin in and bicyclol, N-butylphthalide (NBP) is the third original drug with independent intellectual property rights in China, which was approved by the China Food and Drug Administration, and the first drug from China to advance to the phase II clinical trials of the United States Food and Drug Administration for ischemic stroke. Additionally, NBP, as one of the first batches of indigenously produced new first-class and recommended drugs in the Chinese guidelines for preventing ischemic stroke, was listed in 2010 and 2014 and has become an important adjuvant drug in the first-line therapy of ischemic stroke in China. Dl-3-NBP is the main active component of NBP which was first isolated from celery seeds. Dl-3-NBP is a lipid-soluble drug with good blood-brain barrier permeability. In animal models of ischemic stroke, it has been investigated primarily for its role in improving microcirculation, inhibiting platelet aggregation, resisting oxidative stress, protecting mitochondrial function, alleviating inflammatory reaction, mediating autophagy of nerve cells, promoting neurogenesis and targeting, reducing thrombosis, neuronal apoptosis, and the volume of cerebral infarction in several pathophysiological mechanisms, which reflected its obvious neuro protective effect.^[12–21]^ Previous studies^[22–25]^ have shown that NBP has significant rehabilitation effects on motor and behavioral functions after ischemic stroke based on the efficacy measures of the National Institutes of Health Stroke Scale (NIHSS) and activities of daily living (ADL) or Modified Rankin Scale (mRS) scores, although few studies have involved psychological aspects; recent trials have confirmed that NBP imparts beneficial effects, not only in cerebral ischemia, but also in vascular dementia, Parkinson disease, Alzheimer disease, amyotrophic lateral sclerosis, and delayed encephalopathy.^[26–30]^

Therefore, from the wholistic perspective of the human body, we conducted this research using a sequential therapy of NBP for acute stroke to adopt the NIHSS,^[31]^ ADL,^[32]^ mRS,^[33]^ the Hamilton Anxiety Scale (HAMA),^[34]^ and the Hamilton Depression Scale (HAMD)^[35]^ to explore whether the 90-day NBP sequential therapy could effectively improve physical movement, life activities, and psychological disorders in acute stroke patients.

## 2 Methods

### 2.1 Recruited patients

The purpose and design of our study passed the official evaluation and was approved by the Ethics Committees of the Heze Municipal Hospital and the First Affiliated Hospital of Guangxi Medical University. Written informed consent was obtained from all participants, and our study was conducted in accordance with the Declaration of Helsinki; if a patient died, consent was required from the patient's parents or children (the legal age of adulthood in China is 18 years). This clinical trial was performed between March 2020 and January 2021.

### 2.2 Inclusion criteria

Participants were selected based on the following inclusion criteria: those aged 40 to 80 years; with a clinical diagnosis of acute ischemic stroke that had commenced within 48 hours before enrolment into the study; with a score between 5 and 15 on NIHSS because patients with severe stroke more frequently need skilled care, which may be beyond the scope of our treatment program; with cerebral computed to mographyscan (required to exclude patients who had intracranial hemorrhage); without impaired communication or cognitive function (Mini-Mental State Examination^[36]^ >15) before onset.

### 2.3 Exclusion criteria

Participants were excluded from the study based on the following criteria: those with transient ischemic attack; with a history of depression, psychosis, or severe substance abuse; who received thrombolytic or acupuncture therapy within the previous 3 months; with difficulty swallowing; with cardiogenic embolism and anticipated revascularization of cerebral arteries; with history of stroke; with severe liver or renal dysfunction; with blood coagulation disorders; participating in other clinical trials; who were pregnant or breastfeeding during the study.

### 2.4 Intervention

Patients were randomly assigned to one of the 2 treatment groups with a double-blind, double-dummy design: the on-site investigator called into an automated system that randomly assigned a number corresponding to a medication kit stored at the research site, and the medication in the kit was administered to the patient. The NBP injection and soft capsule, together with the matching placebo, were purchased from Shijiazhuang Pharmaceutical Group Limited Company, which had no other role in the study; all participants received clopidogrel (75 mg once daily on days 1–90) and aspirin (100 mg once daily on days 1–90).

In the experimental group, NBP injection 100 mL intravenous drip was administered twice daily from days 1 to 14, followed by NBP soft capsules 200 mg 3 times daily from days 15 to 90. While in the control group, NBP placebo injection 100 mL intravenous drip was administered twice daily from days 1 to 14, followed by NBP placebo soft capsules 200 mg 3 times daily from days 15 to 90.

The sequential treatment of NBP injection and soft capsules lasted for 90 days. After day 90, treatment choice was dependent on the clinician and patient.

### 2.5 Outcome measures

#### 2.5.1 Primary outcomes

Primary outcomes were ADL^[32]^ and mRS, which were evaluated on day 0 (baseline), day 14, and at 3 and 6 months. The total ADL score was set at 100, and the higher the score, the better the abilities of the daily life of the patients. The total mRS score was set at 6, with a range from 0 (indicating no residual symptoms) to 5 (indicating bed bound, requiring constant care) and 6 (death).

#### 2.5.2 Secondary outcomes

Secondary outcomes were NIHSS, HAMA, and HAMD, which were evaluated on day 0 (baseline), day 14, and month 1, and combined with the application of the ADL^[32]^ and mRS which could initially screen for various neurological and behavioral functions.

The psychological status of all patients was assessed by HAMA and HAMD on day 0 (baseline), and at 3 and 6 months. Moreover, cognitive, language, and functional impairment in patients with acute stroke can make it difficult to recognize PSD and PSA; therefore, the degrees of HAMA and HAMD were both set at moderate to severe (HAMA >21 and HAMD >20) to improve the specificity of psychiatric and reduce the false positive rate, and higher scores indicated worse psychiatric disorder of the patients.

#### 2.5.3 Safety outcomes

The adverse reactions of NBP or other serious adverse events, including primary intracranial hemorrhage, subcutaneous hemorrhage, nasal and gum bleeding, orgastrointestinal hemorrhage, were evaluated at each time point.

### 2.6 Statistical analysis

Our study was a randomized, double-blind, controlled trial, and all data were analyzed using SPSS (version 22.0; SPSS Inc, Chicago, IL) and tabulated using Microsoft Office software (Word, Excel). All continuous variables were expressed as mean ± standard deviation, and Student *t* test and chi-square test were applied to investigate the differences between the 2 groups regarding the demographics and baseline clinical variables (age, sex, body mass index, education level, acute physiology, and chronic health evaluation II,^[37]^ Mini-Mental State Examination and mental labor,^[38]^ single/widowed/divorced, current/previous smoking, hypertension, hypercholesterolemia, diabetes, coronary heart disease, nitrate, antihypertensive agents, diuretics, beta-blockers, calcium antagonists, angiotensin II receptor blockers, angiotensin-converting enzyme inhibitors, lipid-lowering drugs, fibrin, nicotinic acid, statins, antidiabetic drugs, oral hypoglycemic agents, insulin, and platelet counts). We performed an ANCOVA to assess the effect of the treatment modalities on the posttreatment NIHSS, ADL, mRS, and the incidence of HAMA and HAMD; all procedures were 2-tailed, and a value of *P* < .05 was considered statistically significant for all tests.

## 3 Results

### 3.1 Sample size

**Figure 1:**
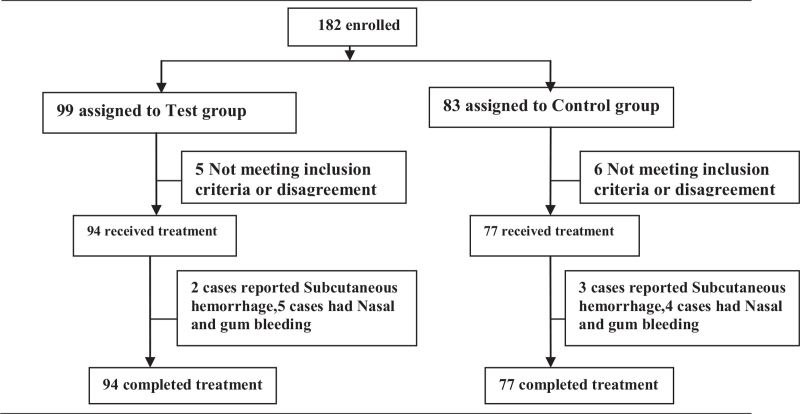
Profile of the clinical trial.

As shown in Figure 1, a total of 182 patients with ischemic stroke were recruited at the start of the study. During the treatment, 5 participants in the control group and 6 in the experimental group were lost to follow-up. Ultimately, 171 patients completed the entire treatment.

There were 77 (45.0%) and 94 (55.0%) patients in the control and experimental groups, respectively. There were no significant differences in any baseline data, such as age, gender, body mass index, educational level, work type, marital status, Chronic health evaluation II, Mini-Mental State Examination, clinical characteristics, concomitant E drugs, and platelet counts. The detailed information is shown in Table 1.

**Table 1:** Baseline characteristics.

Characteristic	Experimental group (n = 94)%	Control group (n = 77)%	Hazard ratio (95% CI)	*P* value
Demographic characteristics				
Age, yrs (Median ± SD)	62.88 ± 8.529	63.21 ± 9.574	–2.410 to 3.060	.815
Range	40-80	40-80		
Female, n (%)	45 (47.9%)	28 (36.4%)	0.622 (0.336–1.152)	.162
Body mass index	22.03 ± 2.609	22.43 ± 2.922	–0.439 to 1.232	.350
Education level (y)	9.91 ± 2.593	10.00 ± 2.601	–0.703 to 0.873	.831
^∗^Mental labor, n (%)	29 (30.9%)	29 (37.7%)	0.738 (0.391–1.394)	.417
Single/widowed/divorced	22 (23.4%)	21 (27.3%)	1.227 (0.614–2.453)	.598
Chronic health evaluation II	14.30 ± 5.408	14.36 ± 5.434	–1.579 to 1.710	.937
Mini-Mental State Examination	21.55 ± 2.530	21.01 ± 3.451	–1.444 to 0.364	.240
Platelet counts (×10^9^)	223.29 ± 59.513	220.27 ± 56.194	–14.598 to 20.627	.827
Selected clinical characteristics				
Current/previous smoking	53 (56.4%)	40 (51.9%)	1.196 (0.653–2.190)	.644
Hypertension	39 (41.5%)	37 (48.1%)	0.767 (0.418–1.406)	.440
Hypercholesterolemia	52 (55.3%)	45 (58.4%)	0.880 (0.479–1.619)	.757
Diabetes	12 (12.8%)	16 (20.8%)	0.558 (0.246–1.265)	.213
Coronary heart disease	19 (20.2%)	11 (14.3%)	1.520 (0.674–3.427)	.419
Concomitant medications				
Nitrates	19 (20.2%)	11 (14.3%)	1.520 (0.674–3.427)	.419
Antihypertensive agents				
Diuretics	21 (22.3%)	20 (26.0%)	0.820 (0.406–1.657)	.594
Beta-blockers	17 (18.1%)	11 (14.3%)	1.325 (0.580–3.028)	.540
Calcium antagonists	31 (33.0%)	32 (41.6%)	0.692 (0.370–1.292)	.268
Angiotensin II receptor blockers	14 (14.9%)	14 (18.2%)	0.788 (0.350–1.772)	.679
Angiotensin-converting enzyme inhibitors	14 (14.9%)	15 (19.5%)	0.723 (0.325–1.610)	.540
Lipid-lowering drugs				
Fibrates	19 (20.2%)	11 (14.3%)	1.520 (0.674–3.427)	.419
Nicotinic acid	15 (16.0%)	16 (20.8%)	0.724 (0.332–1.579)	.432
Statins	38 (40.4%)	36 (46.8%)	0.773 (0.421–1.420)	.440
Antidiabetic drugs				
Oral hypoglycemic agents	10 (10.6%)	14 (18.2%)	0.536 (0.223–1.285)	.187
Insulin	4 (4.3%)	8 (10.4%)	0.383 (0.111–1.325)	.140

PSD = poststroke depression.

∗ Mental labor^[38]^: Mental labor is opposite to physical labor. Labor based on mental consumption. It is characterized by the use of intelligence, scientific and cultural knowledge, and production skills; therefore, it is also called “intellectual labor”. People who exercise mental labor have richer cognitive activity, which is an important risk factor for PSD.

### 3.2 Treatment efficacy

At day 14, and at 3 and 6 months after the treatment, the ADL scores were all significantly higher than those in the control group (53.72 ± 8.948 vs 49.48 ± 7.805; 67.45 ± 12.109 vs 58.64 ± 9.306; 85.85 ± 10.540 vs 80.13 ± 13.002; all *P* < .001), while the mRS scores were all significantly lower than those in the control group (3.22 ± 0.571 vs 3.40 ± 0.494; 2.64 ± 0.526 vs 3.06 ± 0.439; 1.57 ± 1.042 vs 1.99 ± 0.803; all *P* < .05).

The NIHSS scores were significantly lower in the experimental group than those in the control group (11.90 ± 2.379 vs 12.73 ± 2.315; 6.59 ± 3.244 vs 7.65 ± 3.417; all *P* < .05) on days 14, and at 1 month after treatment. Moreover, compared to the control group, the incidence of HAMA was markedly decreased in 15 (16.0%) cases on month 3, and 17 (18.1%) on month 6 after treatment (all *P* < .05). Moreover, there were 9 (9.6%) cases of HAMD on month 3, and 14 (14.9%) on month 6, which were all significantly lower than those in the control group after treatment (all *P* < .05). The detailed information is shown in Table 2.

**Table 2:** Comparison of the NIHSS, ADL, mRS and HAMA, HAMD at different time points in 2 groups.

	Experimental group (N = 94)	Control group (N = 77)	*P* value
Primary outcomes			
ADL			
Before treatment	34.36 ± 6.402	33.51 ± 5.853	.368
On the days 14	53.72 ± 8.948	49.48 ± 7.805	.001
On the months 3	67.45 ± 12.109	58.64 ± 9.306	<.001
On the months 6	85.85 ± 10.540	80.13 ± 13.002	.002
mRS			
Before treatment	3.98 ± 0.145	3.96 ± 0.195	.498
On the days 14	3.22 ± 0.571	3.40 ± 0.494	.031
On the months 3	2.64 ± 0.526	3.06 ± 0.439	<.001
On the months 6	1.57 ± 1.042	1.99 ± 0.803	.005
Secondary outcomes			
NIHSS			
Before treatment	17.07 ± 2.263	17.10 ± 2.326	.934
On the days 14	11.90 ± 2.379	12.73 ± 2.315	.024
On the month 1	6.59 ± 3.244	7.65 ± 3.417	.039
HAMA			
On the months 3	15 (16.0%)	26 (33.8%)	.007
On the months 6	17 (18.1%)	29 (37.7%)	.005
HAMD			
On the months 3	9 (9.6%)	19 (24.7%)	.012
On the months 6	14 (14.9%)	25 (32.5%)	.010
Safety outcomes			
Primary intracranial hemorrhage	0	0	
Gastrointestinal hemorrhage	2 (2.1%)	3 (3.9%)	.659
Nasal and gum bleeding	5 (5.3%)	4 (5.2%)	1.000
Subcutaneous hemorrhage	0	0	

ADL = Barthel Index of activities of daily living, HAMA = Hamilton Anxiety Scale, HAMD = Hamilton Depression Scale, mRS = Modified Rankin Scale, NIHSS = National Institutes of Health Stroke Scale.

### 3.3 Adverse events

There were no significant alterations in blood glucose, blood lipids, or liver and kidney function during the whole treatment session. As shown in Table 2, no primary intracranial hemorrhage or subcutaneous bleeding was observed in either group. There were 2 (2.1%) cases with gastrointestinal hemorrhage, 5 (5.3%) cases with nasal and gum bleeding in the test group, 3 (3.9%) cases with gastrointestinal hemorrhage, and 4 (5.2%) cases with nasal and gum bleeding in the control group. However, these patients with side effects including primary intracranial hemorrhage, subcutaneous hemorrhage, nasal and gum bleeding, and gastrointestinal hemorrhage had spontaneous remission and needed no additional medications. There was no significant difference in the incidence of bleeding between the 2 groups.

## 4 Discussion

In our study, our therapy could significantly increase the ADL scores, decrease the mRS and NIHSS scores, and the incidence of HAMA and HAMD in ischemic stroke patients after acute-phase treatment, which could significantly improve the neurological and behavioral function outcomes and decrease psychological disorders in acute ischemic stroke patients. Moreover, as the ADL increased, the mRS and incidence of HAMA and HAMD decreased significantly in the experimental group, suggesting that the addition of NBP in the course of disease management has a better effect in protecting and restoring nerve function. Moreover, the addition of NBP did not significantly increase the incidence of adverse events. Therefore, 90 days of NBP sequential therapy combined with clopidogrel and aspirin in the treatment of ischemic stroke can achieve better clinical efficacy and is expected to become a potential new treatment method for middle-aged and elderly acute ischemic stroke patients although it requires further exploration.

Xu et al^[22]^ searched major databases to identify randomized controlled trials that assessed the efficacy and safety of NBP on ischemic stroke, reporting outcomes among patients treated with NBP alone or combined with standard anti-ischemic stroke drugs vs standard anti-ischemic stroke drugs alone; the combined use of NBP and standard anti-ischemic stroke drugs is more effective than standard drugs alone. Xue et al^[23]^ suggested that a 10-day treatment with NBP or cerebrolysin can be applied safely and may provide beneficial effects for acute ischemic stroke patients, particularly for cases with moderate severity, and NBP appears to be more effective than cerebrolysin in improving the short-term prognosis of acute ischemic stroke patients. Their findings provided established evidence of NBP as a neuroprotective drug, which may improve the current guidelines for the treatment of ischemic stroke.

Previous studies^[22–24]^ have shown that NBP has significant rehabilitation effects on motor and behavioral functions after ischemic stroke, although few studies have addressed the psychological aspects. However, a decrease in stroke lethality along with the aging of the population and epidemiological transition to chronic non-infectious diseases leads to an increasing number of stroke survivors. PSD occurs in approximately one-third of all stroke survivors.^[39]^ PSD patients usually present with extensive symptoms, including weight changes, apathy, feelings of worthlessness, sleep disturbances, and fatigue.^[40]^ There has been sufficient documentation that PSD reduces the quality of life and impedes rehabilitation in most stroke survivors.^[41]^ PSD is a complex multifactorial process involving various biological, behavioral, and social factors.^[42]^ However, to date, the pathophysiological mechanism of PSD has not been fully elucidated. In addition to its high incidence and prevalence, previous studies have reported several risk factors for PSD, including sex, age, lesion location, stroke severity, functional disability,^[43–46]^ poorer cognitive activity, impaired functional rehabilitation, and poorer quality of life, as well as higher mortality, which may be up to 10 times higher than that in patients without PSD.^[41,47,48]^ Therefore, it is critical to develop an effective antidepressant treatment for this specific population to alleviate neurological deficits and facilitate stroke rehabilitation. Previous studies indicate that the correlation between PSD and stroke severity, physical disability, and cognitive impairment is the most consistent.^[49]^ In 2014, Kutlubaev et al^[50]^ observed that stroke severity and poststroke physical disability were consistent with depression. Therefore, solving physical disabilities may be the key to the treatment of PSD.

In China, NBP was approved by the China Food and Drug Administration as the third original drug with independent intellectual property rights and one of the first batch of new national first-class drugs and the recommended drugs in Chinese guidelines for preventing ischemic stroke. It was listed in 2010 and 2014 and is currently becoming an important adjuvant drug in the first-line therapy of ischemic stroke. Both Cui et al and Wo et al^[24,25]^ used NBP sequential therapy combined with dual antiplatelet therapy in the treatment of acute cerebral infarction in China. Using the changes in the NIHSS and ADL or mRS scores as measures of efficacy, NBP sequential therapy combined with dual antiplatelet therapy was effective in the treatment of acute cerebral infarction in elderly patients. It is conducive to improving the living ability and neurological function of patients and has high safety. Our study showed that the addition of NBP, an original drug with independent intellectual property rights in China, can significantly reduce psychological disorders, which may be related to its ability to directly improve functional disability and indirectly reduce psychological disorders in patients.

NBP, an emerging anti-ischemic drug, may be correlated with the mechanism of ischemic stroke with its unique pharmacological effects: inhibiting platelet aggregation and reducing thrombosis^[51–53]^; reversing microvascular spasm, improving cerebral blood flow, and restoring damaged microcirculation^[54]^; inducing brain microangiogenesis, preventing a hypothermic response to ischemic stroke^[55,56]^; improving mitochondrial morphology after ischemia, maintaining mitochondrial membrane fluidity, and protecting mitochondrial potential, thereby reducing cerebral edema^[57]^; inhibiting a series of caspase-dependent and non-caspase-dependent 4 proteins and the activation of c-Jun N-terminal kinases reperfusion-induced neuronal apoptosis^[58,59]^; blocking ischemia, promoting axons, synapsing formation, and nerve regeneration in the subventricular region after stroke,^[60]^ while promoting the proliferation of hippocampal cells, increasing the survival rate of neoplastic cells, and adjusting the differentiation of neoplastic cells to mature neurons after ischemic damage; regulation of the induction of nitric oxide synthase messenger ribonucleic acid levels and inhibition of nuclear factor kappa-light-chain-enhancer of activated B cells activation protects neurons from oxidative stress^[61]^; and preventing neutrophil infiltration, reducing intercellular expression after ischemic injury^[62]^; and reducing astrocyte activation,^[63]^ thereby reducing inflammation and reducing damage after ischemic injury.

### 4.1 Study limitation

Our study had several limitations. First, the sample size was small, as our enrolment did not include all middle-aged or elderly patients with acute ischemic stroke originating from all factors, or severe stroke patients with dysphagia. Second, the follow-up was only 6 months, which was too brief to evaluate the psychology of patients precisely. Further trials are required to evaluate the efficacy of long-term NBP therapy in ischemic stroke, and the differences between doses should also be evaluated regarding the improvement of neurological, behavioral, and psychological outcomes. Third, our study was conducted in only 2 hospitals; thus, further studies are needed to confirm the efficacy and safety of NBP as an additional therapy in the treatment of ischemic stroke. Multicenter, long-term, and large-scale studies with more comprehensive efficacy evaluation systems are required to further verify our conclusions. Due to these conditions, to date, the related factors have not been analyzed and should be considered in future research experiments. However, we believe that our findings are valuable.

## 5 Conclusion

Despite the limitations of our study, our results indicated that 90 days of sequential therapy with NBP as an additional therapy in the treatment of ischemic stroke can better improve patients’ psychological and behavioral functions without significant side effects.

## Acknowledgments

Sincere gratitude is extended to the nurses in our department and participants for their efforts and cooperation.

## Author contributions

Yankun Chen and Jieqiong Du contributed to the conception and design of the study. Yanzhi Wu, Hongdan Zhang, and Hui Li organized the database. Le Yang wrote the first draft of the manuscript. Yankun Chen, Yanzhi Wu, and Hui Li wrote sections of the manuscript. All authors contributed to the manuscript revision, read, and approved the submitted version.

**Conceptualization:** Yankun Chen.

**Data curation:** Le Yang, Hui Li, Yanzhi Wu, Hongdan Zhang, Jieqiong Du, Yankun Chen.

**Formal analysis:** Yankun Chen.

**Investigation:** Le Yang, Hui Li, Jieqiong Du, Yankun Chen.

**Methodology:** Le Yang, Yankun Chen.

**Project administration:** Yankun Chen.

**Resources:** Le Yang, Hui Li, Jieqiong Du.

**Software:** Le Yang, Yankun Chen.

**Supervision:** Le Yang, Hui Li, Jieqiong Du.

**Validation:** Le Yang.

**Visualization:** Hui Li, Yankun Chen.

**Writing – original draft:** Le Yang.

**Writing – review & editing:** Yankun Chen.
